# Considerations of complexity in rating certainty of evidence in systematic reviews: a primer on using the GRADE approach in global health

**DOI:** 10.1136/bmjgh-2018-000848

**Published:** 2019-01-25

**Authors:** Paul Montgomery, Ani Movsisyan, Sean P Grant, Geraldine Macdonald, Eva Annette Rehfuess

**Affiliations:** 1 School of Social Policy, University of Birmingham, Birmingham, UK; 2 Centre for Evidence-Based Intervention, Department of Social Policy and Intervention, University of Oxford, Oxford, UK; 3 Pardee RAND Graduate School, RAND Corporation, Santa Monica, California, USA; 4 School for Policy Studies, University of Bristol, Bristol, UK; 5 Institute for Medical Information Processing, Biometry and Epidemiology, Pettenkofer School of Public Health, Ludwig Maximilian University, Munich, Germany

**Keywords:** systematic review, public health

## Abstract

Public health interventions and health technologies are commonly described as ‘complex’, as they involve multiple interacting components and outcomes, and their effects are largely influenced by contextual interactions and system-level processes. Systematic reviewers and guideline developers evaluating the effects of these complex interventions and technologies report difficulties in using existing methods and frameworks, such as the Grading of Recommendations Assessment, Development and Evaluation (GRADE). As part of a special series of papers on implications of complexity in the WHO guideline development, this paper serves as a primer on how to consider sources of complexity when using the GRADE approach to rate certainty of evidence. Relevant sources of complexity in systematic reviews, health technology assessments and guidelines of public health are outlined and mapped onto the reported difficulties in rating the estimates of the effect of these interventions. Recommendations on how to address these difficulties are further outlined, and the need for an integrated use of GRADE from the beginning of the review or guideline development is emphasised. The content of this paper is informed by the existing GRADE guidance, an ongoing research project on considering sources of complexity when applying the GRADE approach to rate certainty of evidence in systematic reviews and the review authors’ own experiences with using GRADE.

**Key questions**

**What is already known?**

- The Grading of Recommendations Assessment, Development and Evaluation (GRADE) approach is an internationally prominent system for rating certainty of the evidence in systematic reviews estimating intervention effects.
- Researchers conducting systematic reviews on public health and health system interventions report difficulties in using the GRADE approach.

**What are the new findings?**

- Applying a ‘complexity perspective’ can help identify aspects of using the GRADE approach that require particular consideration when rating certainty in the estimates from systematic reviews estimating the effects of global health interventions.
- These aspects include: sources of complexity when framing the review questions, such as important dimensions of context and implementation and other potential mediators and moderators of effect; a choice of thresholds or ranges for certainty of evidence ratings that matches the needs of intended users of the review; assessment of evidence from non-randomised study designs; the criteria within each GRADE domain for rating certainty of evidence and coherence of evidence across the hypothesised causal pathway of the intervention.

**What do the new findings imply?**

- This primer can help systematic reviewers, health technology assessors and guideline developers better assess evidence relating to complex interventions and systems, which could enhance the use of such evidence in global health policy and practice decisions.

## Introduction

### Systematic reviews on the effects of interventions in global health

Systematic reviews that estimate the effects of interventions can have a significant influence on subsequent decisions to either implement or disinvest in an intervention. In biomedicine, there are established methods for synthesising and rating certainty in the effects of medications and other single-component interventions.^1^ Researchers in public health and health technology assessment (HTA) report difficulties in using these methods,^2 3^ largely stemming from failure to account for ‘what interventions work, for whom, and under what circumstances’,^4^ to further inform development of context-specific recommendations.^5 6^

WHO is the leading institution for producing evidence-informed guidelines at a global level. WHO recommendations for practice and policy are underpinned by systematic reviews of evidence on the effects of interventions and health technologies and aim to follow a transparent and evidence-based process.^7^ Review and HTAs that inform recommendations often need to consider a range of populations, interventions with different implementation strategies, multiple health and non-health outcomes and various contextual factors that may interact and modify intervention effects.^8^ Given the pressing needs and limited resources in low-income and middle-income countries (LMICs), appropriate application of evidence synthesis on a broad range of health interventions is crucial for optimal decisions about implementation.^9^

### Using a complexity perspective in reviews of intervention effects

Recently, there has been increased attention to evaluating effects of complex health interventions implemented within complex systems.^10^ This complexity creates challenges for guideline development and HTAs, from the conceptualisation of the questions asked to synthesising diverse types of evidence, assessing or rating the evidence and formulating recommendations. This paper is one of a series exploring the implications of complexity for systematic reviews, HTAs and guideline development.

Using a complexity perspective in systematic reviews of the effects of interventions can facilitate the more nuanced conceptualisation and assessment of interventions ultimately needed for health decision making.^11^ An earlier paper in this series differentiates between two main perspectives on sources of complexity in the evaluation of interventions.^10^ A ‘complex interventions perspective’ locates sources of complexity in the features of interventions themselves, such as interventions with different components addressing different and multiple causes of problems.^12^ A ‘complex systems perspective’, on the other hand, locates sources of complexity in the properties of systems into which interventions are introduced, such as how the intervention interacts with, and impacts on, the system as a whole.^13^ We use the overarching term ‘complexity perspective’ to encompass both perspectives and acknowledge the many sources of complexity.^10^ Depending on the priority questions of a review, an HTA, or a guideline, either perspective or a combination may be adopted.

In planning and undertaking systematic reviews, a ‘complex systems perspective’ necessarily entails broadening the scope of a review to include evidence on how the wider system changes when the intervention, such as a complex technology, is introduced. This may involve collecting qualitative evidence on social norms and the dynamics of social networks to describe the broader system impact of the intervention. Not all sources of complexity are relevant to every systematic review and HTA. Researchers should take a pragmatic approach that focuses on the key aspects of interventions, their causal pathways and the levels of target relevant to the specific aims of the review and users’ needs. There is a growing body of literature and guidance, which can be helpful in deciding on the important sources of complexity to consider in systematic reviews, HTAs and guidelines, including approaches described in earlier papers in this series, by Booth *et al,*^14^ Petticrew *et al*^10^ and Rehfuess *et al*^15^ (see box 1).

Box 1 **Examples of guidance and tools for addressing sources of complexity**

- Context and Implementation of Complex Interventions framework^63^
- Guidance on the integrated assessment of complex health technologies: the INTEGRATE-HTA model^64^
- Intervention Complexity Assessment Tool for Systematic Reviews^65^
- Preferred Reporting Items for Complex Interventions for Systematic Reviews and Meta-analyses^66^
- Template for Intervention Description and Replication tool^6 67^

### The GRADE approach to rating certainty of evidence

The GRADE Working Group has taken a leading role in developing guidance and methods for using research evidence to inform healthcare recommendations. Grading of Recommendations Assessment, Development and Evaluation (GRADE) offers an explicit and transparent system for rating certainty in the body of evidence underpinning conclusions in a systematic review, an HTA or a guideline (box 2). In GRADE, certainty of the effect estimate for each outcome is ultimately assigned one of four categories: high, moderate, low or very low. The GRADE approach has been widely adopted by systematic reviewers, authors of HTA and guideline developers in healthcare, including over 100 organisations worldwide.^16^ Among these, WHO uses GRADE to inform global health recommendations,^16^ and the Cochrane Collaboration mandates use of GRADE in Cochrane intervention reviews.^17^

Box 2 **Summary of the GRADE process for rating the certainty of evidence for intervention effects**

- The Grading of Recommendations Assessment, Development and Evaluation (GRADE) process starts with an initial certainty rating based on the design of studies included in the body of evidence: if the body of evidence contributing to an outcome consists of randomised controlled trials, certainty is initially rated as ‘high’, whereas a body of evidence consisting of observational or non-randomised studies (NRSs) is initially rated as ‘low’.
- The assessing team then uses five domains for potential downgrading of the initial certainty rating: study limitations, indirectness, inconsistency, imprecision and publication bias.
- Next, the team assesses three further domains for potential upgrading of the initial certainty rating: magnitude of the effect, dose–response relationship in the effect and counteracting plausible residual bias or confounding.^43^ These upgrading domains are primarily relevant to NRSs (eg, cohort, before–after and interrupted time series).
- Evidence Profiles and Summary of Findings tables are used to summarise the effect estimates and the certainty ratings for those estimates for each main outcome in the assessment.
- The GRADE ratings are further used as one of the criteria in the Evidence to Decision frameworks to inform recommendations about implementing interventions in practice, where high-certainty evidence is more likely to result in a strong recommendation compared with low-certainty evidence.^68^

Despite its wide uptake in biomedicine, systematic reviewers and guideline developers report difficulties applying GRADE in reviews of broader health technologies, health system and public health interventions.^2 3^ These challenges are frequently attributed to the complexity of these interventions, often requiring sophisticated consideration and analysis.^3 13^ For example, high levels and various sources of heterogeneity in reviews of public health interventions often lead to challenges in deciding how and whether to downgrade for inconsistency.^3 18^ Another common challenge results from the difficulty, if not impossibility, of using RCTs to evaluate policy-level and health system interventions.^2 3^ With all types of non-RCT evidence starting off at a ‘low’ certainty level, public and global health researchers have voiced concerns that GRADE may inadvertently produce ratings that steer decision-makers away from implementing important system-level interventions.^19–21^ Concerns have also been raised on how to conceptualise the construct of ‘certainty’ in reviews of global health interventions, and consensus is currently lacking.^3^ Consequently, global health researchers could benefit from targeted guidance on how to rate certainty when encountering these challenges.^2 3 9 21 22^

### Objectives

As part of the overall series intended to stimulate thinking about how methods for reviewing and assessing evidence in guideline development can be enhanced to take account of complexity, this paper clarifies how a complexity perspective may be applied when using the GRADE approach to rate certainty of evidence. The GRADE Working Group is actively working to advance the GRADE methodology for different applications, including for diagnostic tests, prognostic studies and qualitative evidence.^16 23^ This paper focuses on using the GRADE approach for rating certainty in the evidence from systematic reviews *estimating the effects* of complex interventions and technologies in global health.

## Methods

This paper is largely informed by an ongoing, mixed-methods research project, *GRADE Guidance for Complex Interventions,* involving five key studies that follow an established methodology on developing guidance for health research.^24^ In Study 1, we investigated GRADE certainty ratings in 24 ‘complex’ and 16 ‘simple’ systematic reviews^18^ and obtained feedback from review authors on 19 of these reviews about their process of applying GRADE.^2^ In Study 2, we compared domains and criteria across GRADE and 16 other systems for rating certainty of evidence in health and social interventions.^25^ In Study 3, we interviewed 10 Cochrane review authors and 5 GRADE methodologists on their views about the challenges of, and suggestions for, using GRADE in specific systematic reviews incorporating various sources and degrees of intervention and system complexity (Movsisyan *et al*, forthcoming). In Study 4, we conducted an online modified-Delphi process to explore areas of agreement and disagreement among 116 stakeholders about the importance of specific domains and criteria for rating certainty in systematic reviews of complex interventions (Grant *et al*, forthcoming). In Study 5, we held a 3-day consensus meeting to discuss proposals for the content of the new GRADE guidance for complex interventions with 28 stakeholders, purposively invited from the Delphi process, representing a range of subject areas (Movsisyan *et al*, forthcoming). All studies were approved by the Departmental Research Ethics Committee at the Department of Social Policy and Intervention, University of Oxford (SPI_C1A_16_009). This project draws suggestions from several sources–including the existing GRADE guidelines and conceptual papers, previous work considering complexity in systematic reviews, HTAs and guideline development and consultation with relevant stakeholders–to advise how to apply the GRADE approach using a complexity perspective in the context of global health.

## Results

Several aspects of using GRADE require particular consideration when using a complexity perspective in systematic reviews and HTAs on the effects of interventions in global health (see table 1). Particularly important is that authors consider GRADE from the outset of the review or HTA and not at the end when evidence has already been synthesised. In this way, the totality of the evidence will become an integral part of the assessment from its inception. As systematic reviews represent an important source of evidence and are integral to most HTAs and guidelines in global health, below we describe how specific constructs and domains of GRADE can be used in systematic reviews using a complexity perspective.

**Table 1:** Mapping the main sources of complexity onto difficulties in rating estimates of the effect of interventions (data taken from Movsisyan *et al*, 2016; Petticrew *et al*, 2013; Petticrew *et al*, 2019; Rehfuess and Akl, 2013)^2 3 10 69^

Source of complexity	Difficulties in rating estimates of the effect of interventions
Multiple components	Interventions are comprised of different components, which may interact (synergistically or dysynergistically) Need to assess the effects of interventions as bundles or specific intervention components
Flexibility or tailoring or non-standardisation of implementation	Ambiguities around how to assess fidelity to intervention implementation
Long causal pathways	Lack of direct evidence linking interventions with distal outcomes Need to integrate different pieces of evidence from potentially different bodies of evidence to estimate the distal effects
Effects are contingent on recipients’ and providers’ agency	It may be impossible to blind recipients and providers of interventions
Multiple outcomes	Need to prioritise between a range of important (health and non-health) outcomes
Effects at different levels, for example, individual and population levels	Need to consider outcomes at different levels (eg, individual, family and societal levels) Population-level interventions are frequently impossible to evaluate using RCTs, which results in downgrading the ‘best evidence possible’ for these interventions because of initial categorisation of evidence in GRADE based on study design
Moderating effects of context	Need to account for various implementation and contextual factors, when conceptualising and rating estimates of the effect

GRADE, Grading of Recommendations Assessment, Development and Evaluation; RCT, randomised controlled trial.

### Considering complexity and GRADE when framing the question(s) and conducting the systematic review(s)

The certainty ratings at the final stage of the review are inextricably linked to the purpose and key questions established at the beginning of each complex intervention review. Similar to the approach described in Petticrew *et al,* 2019^10^, reviewers and guideline developers should identify sources and degrees of complexity inherent in interventions themselves, as well as the systems in which they are implemented and intended to influence (see table 1). As emphasised in the WHO-INTEGRATE framework (see Rehfuess *et al*, 2019^15^), incorporation of sources of complexity into the review and, ultimately, into the GRADE ratings should be considered at the earliest stages of the review process: explicitly addressing sources of complexity when formulating review questions^14^ ^15^ and structuring the proposed GRADE Evidence Profiles and Summary of Findings (SoFs) tables.^26^ Thinking through all relevant sources of heterogeneity at the beginning of the systematic review process will influence the types of data extracted and syntheses conducted. For instance, for a Cochrane review of environmental interventions to reduce the consumption of sugar-sweetened beverages and their adverse effects on health,^27^ reviewers developed a system-based logic model to guide data extraction, analysis and interpretation. The frameworks to extract and report relevant data were prespecified as were internal and external sources of heterogeneity for subgroup analysis (box 3). Such an approach is essential for capturing heterogeneity in the methodology and Population, Intervention, Comparison, Outcome (PICO) elements, which would otherwise remain unexplained and almost inevitably lead to downgrading of evidence.

Box 3 **Consideration of sources of complexity in a protocol for a Cochrane review of environmental interventions to reduce consumption of sweetened beverages (von Philipsborn *et al*)^27^**

von Philipsborn *et al* (2016) developed a system-based logic model taking into account:

- Beverage choices and diet-related health and non-health outcomes
- Physiological and psychological mechanisms linking sugar-sweetened beverages with health outcomes at an individual level
- Interventions aimed at policy (macro) and settings (meso) and interpersonal and intrapersonal factors (micro level)
- Determinants of diet-related outcomes and related interventions

This logic model was used to guide data extraction, analysis and interpretation.

Tools used for data collection:

- Template for Intervention Description and Replication framework to extract and report relevant data related to the intervention^6^
- Context and Implementation of Complex Interventions framework for contextual data^63^

Internal and external sources of heterogeneity were predefined at multiple levels, such as at policy or setting level:

- With or without behavioural cointerventions
- Targeted at: sugar-sweetened beverages, sugar-sweetened milk, beverages with non-nutritive sweeteners or beverages without added sweeteners
- Implemented in high-income, middle-income or low-income countries
- Targeted at the general population or at disadvantaged populations

A frequent challenge for authors of intervention reviews in global health arises from posing broad review questions on bundles of conceptually similar interventions (often referred to as a ‘lumping’^28^ or a ‘holistic'^29^ approach) that may actually have very different characteristics.^30 31^ In these cases, authors should carefully consider upfront which sources of complexity are critical to include in their research questions, such as the active or ‘prototypical’ components of an intervention that are most likely to modify intervention effects.^32^

Logic models may be particularly helpful in depicting intervention components and identifying potential effect modifiers.^10 11 32 33^ A recent example involves a systematic review by Welch *et al* (2016) aiming to estimate the effects of deworming interventions on the developmental health and well-being outcomes of children in LMICs.^34^ While evidence for deworming programmes had been debated,^35–37^ review authors made considerable efforts to describe the complexities of the programme. Specifically, they developed a logic model at the outset of the review to elucidate the entire causal chain from worm infection to nutritional status and educational outcomes, how deworming–in combination with other strategies (such as hygiene promotion and sanitation)–intervenes in the pathway and which factors might be important in mediating or moderating the effects (such as poverty, prevalence and intensity of infection and spill-over effects).^34^ This complexity perspective allowed them to add important questions to their overall question of ‘what works’, such as the effects of deworming according to the prevalence of infections, as well as the synergistic effects of cointerventions and treatment externalities for untreated children. They then constructed three separate GRADE SoF tables to provide certainty ratings depending on the levels of endemicity for which different strategies of mass deworming were relevant.^34^

### Defining the thresholds or the ranges for certainty of evidence ratings

The GRADE Working Group conceptualises ‘certainty of evidence’ as confidence that the true effect of an intervention lies on one side of a specified threshold or within a chosen range (see table 2).^38^ In general, depending on the purpose of the assessment (ie, whether the systematic review informs a guideline or not), certainty of evidence ratings are presented as ‘non-contextualised’, ‘partly contextualised’ and ‘fully contextualised’. Non-contextualised ratings are relevant for assessments conducted outside of a guideline (eg, Cochrane and Campbell reviews): in these circumstances, authors may prioritise the threshold of the null effect and conceptualise certainty of evidence as confidence that a non-null effect is present, that is to say, that the effect of one intervention differs from another. Alternatively, the range approach may be chosen, and certainty of evidence may be conceptualised as confidence that the effect lies within a given range (eg, a 95% CI or prediction interval). Finally, authors may instead choose a partly contextualised rating, setting thresholds of specified magnitudes of effect (eg, what may be considered as a trivial, small, moderate or large effect). Fully contextualised ratings are relevant when systematic reviews are conducted as part of a specific guideline development or decision-making process, which enables integration of other considerations relevant for a health decision. In this case, authors could rate the certainty that the effect lies above a threshold that makes implementation of the intervention worthwhile.^38^

**Table 2:** Approaches for setting thresholds or ranges for certainty of evidence ratings (adapted from Hultcrantz *et al*, 2017)^38^

Setting	Contextualisation	Threshold or range	How to set	What certainty rating represents
Primarily for systematic reviews and health technology assessment	Non-contextualised	Range: 95% CI	Using existing limits of the 95% CI, which implies that precision is not routinely part of the rating	Certainty that the effect lies within the CI
		OR≠1; RR≠1; HR≠1; RD≠0	Using the threshold of null effect	Certainty that the effect of one treatment differs from another
Primarily for systematic reviews and health technology assessment	Partly contextualised	Specified magnitude of effect	For example, small effect is the effect small enough to not use the intervention if adverse effects/costs are appreciable	Certainty in a specified magnitude of effect for one outcome (eg, trivial, small, moderate or large)
Primarily for practice guidelines	Fully contextualised	Threshold determined with consideration of all critical outcomes	Considering the range of effects on all critical outcomes, and the values & preferences for those ranges	Confidence that the direction of the net effect will not differ from one end of the certainty range to the other

RD, risk difference; RR, risk ratio.

The non-null effect is likely the simplest and most feasible threshold for rating certainty on the effects of public health interventions. Since these intervention effects may vary depending on implementation factors, context and settings, it may be very challenging for a review group to define specific magnitudes of effect for various outcomes that are practically important for *all* potential contexts of application. Rating certainty in the non-null effect would inform the broad global readership about the general direction (positive or negative) of an intervention effect. The task will then be left for the end-users of the evidence at the local level to further contextualise the evidence and set the corresponding thresholds informing implementation of the intervention depending on their specific circumstances. Importantly, contextualising the evidence usually involves a broad range of considerations and decisions not solely driven by evidence of intervention effectiveness.^15^

The choice of the thresholds or ranges will have implications for how the domains of the GRADE approach are applied in a given review or guideline. For example, the criteria of imprecision and inconsistency are only marginally relevant when assessing certainty in the non-null effect (as long as there is consistency in the direction of effect across studies), but are highly relevant when assessing certainty in a specified magnitude of effect. It is therefore critical for systematic reviewers and guideline developers to make their choice explicit and to carry it through into rating the evidence (see table 3 for further details).

**Table 3:** Key considerations for rating certainty in systematic reviews on the effects of complex interventions

Recommendation	Rationale
**Deciding on the scope of the review**	
1. Use logic models to develop PICO and review questions	Logic models help in scoping, defining and conducting the review and in making the review relevant to policy and practice. Approaches have been developed to assist with this^10 33 58 70^
2. Identify which tools to use to best describe the sources of complexity that users will require	There are several newly developed tools on using a complexity perspective in systematic reviews, such as the approach by Petticrew *et al* 2019,^10^ iCAT_SR, the CICI framework, TIDieR and PRISMA-CI^6 63 65 66^
3. Using these tools identify contextual and implementation factors and other moderators of effect that may help explain heterogeneity and which will need separate GRADE certainty ratings	In addition to the standard PICO question, identify in both the intervention and the system in which it is being used all the complexities and interactions that review users will want to know about^10^ Under intervention complexities, consider aspects of its implementation, such as theory of why and how the intervention is expected to work, the components, implementers, mediators, moderators, and causal pathways Under system complexities, consider context, setting and any other independent interventions taking place
**Defining thresholds or ranges for certainty of evidence ratings**	
Define ‘certainty’ in a manner that matches the needs of the intended users of the review	Decide among the three approaches to defining certainty of evidence: ‘non-contextualised’, ‘partly contextualised’ and ‘fully contextualised'^38^ In each case, specify the threshold or ranges used to rate certainty of evidence For ‘non-contextualised’ reviews, consider the utility of using GRADE for the ‘non-null’ effect
3. Using these tools identify contextual and implementation factors and other moderators of effect that may help explain heterogeneity and which will need separate GRADE certainty ratings	In addition to the standard PICO question, identify in both the intervention and the system in which it is being used all the complexities and interactions that review users will want to know about^10^. Under intervention complexities, consider all aspects of its implementation, including theory of why and how the intervention is expected to work, the process, the components, implementers, moderators, causal pathways (linear and non-linear) and important process outcomes Under system complexities, consider context, setting (eg, individual or population level) and any other independent interventions taking place
**Rating certainty of evidence using GRADE**	
1. Initially rate any body of evidence as ‘high’ if a rigorous tool is used to assess risk of bias in NRSs (ie, ROBINS-I), otherwise, use the ‘standard’ GRADE guidance	Consider using Cochrane Risk of Bias (RoB V.2.0) tool for randomised controlled trials^42^ Consider using ROBINS-I for cohort-type studies^41^
2. Give extra scrutiny to the impact of lack of blinding providers/participants on overall risk of bias for outcomes	If lack of blinding of either participants of providers is unlikely to affect assessment of outcome (such as when using objective outcome measures, for example, mortality), then consider not downgrading evidence for lack of blinding for that outcome.
3. Consider the effect of bias associated with deviation from the intended intervention	Deviations, such as poor adherence, poor implementation and cointerventions in relation to the effect of starting and adhering to an intervention, may lead to bias and may be downgraded by one level Consider not downgrading if assessing the effect of assignment to the intervention, when deviations do not occur in relation to usual practice and groups remain balanced
4. Consider multiple criteria for judging inconsistency of evidence	Assessment of heterogeneity should always start off with an appraisal of study heterogeneity, including heterogeneity in PICO elements as well as methodological aspects Assessment of heterogeneity should take account of multiple rather than single criteria for inconsistency (eg, I^2^ and its p value, overlap of CIs and degree of variation within chosen thresholds) Consider whether definition of certainty of evidence influences nature of inconsistency assessment (eg, when effect sizes across all studies are consistently in the same direction outside of the null effect or a given threshold of interest, then downgrading for inconsistency is not warranted despite other measures) Consider different analytical methods to explain heterogeneity (eg, subgroup analysis, meta-regression and qualitative comparative analysis)
5. Rate imprecision of evidence with regard to the adopted definition of ‘certainty’	Consider whether definition of certainty of evidence influences nature of imprecision assessment^38^ For ‘non-contextualised’ systematic reviews definition, a certainty that the effect lies within estimated CIs or prediction intervals, a GRADE assessment for imprecision can usually be omitted as assessment of precision is dependent on the chosen range For ‘partly contextualised’ systematic reviews, consider whether the point estimate would represent a trivial, small, moderate or large absolute effect For ‘fully contextualised’ systematic reviews, simultaneously consider all important outcomes to determine precision of the effect estimate
6. Examine indirectness of evidence by way of assessing important differences in the evidence base beyond what is expected	Consider grouping studies, synthesising evidence and rating certainty in the estimates of effect for separate outcomes according to the relevant sources of complexity identified at the start of the review Consider splitting the questions to answer subset conditions, downgrading only for those with less certain evidence. Do not downgrade for indirectness if observed differences are unlikely to affect the outcome
7. Consider publication bias	Conduct extensive grey literature searches and expert contacts to identify reports and working papers Consider sponsorship of studies by any vested industries as well as potential ‘allegiance bias’
8. Upgrading evidence	Consider upgrading certainty of evidence for a dose–response relationship related to the level of implementation Consider upgrading evidence for a body of evidence from studies with low implementation fidelity positive results which counteract plausible residual bias or confounding
Use logic models to investigate coherence of evidence across the causal pathway	Consider assessing the coherence of evidence across different links in the causal pathway at the end of evidence synthesis. This judgement should be made outside of the GRADE framework

CICI, Context and Implementation of Complex Interventions; GRADE, Grading of Recommendations Assessment, Development and Evaluation; iCAT-SR, Intervention Complexity Assessment Tool for Systematic Reviews; NRS, non-randomised study; PICO, Population, Intervention, Comparison, Outcome; PRISMA-CI, Preferred Reporting Items for Complex Interventions for Systematic Reviews and Meta-analyses; ROBINS-I, risk of bias in non-randomised studies; TIDieR, Template for Intervention Description and Replication.

### Initial certainty rating based on study design

Following definitions of review scope and thresholds or ranges for rating the certainty of evidence, the initial rating is based on study design. Given the practical impossibilities of conducting RCTs for many complex interventions, global health researchers have struggled with the convention that a body of RCTs is initially rated as ‘high’ certainty in the GRADE approach and a body of NRSs as ‘low’ certainty.^2 3 39^ Moreover, there are clearly differences with respect to one’s ability to draw causal inferences about intervention effects from a cross-sectional study, a very weak design for that aim, compared with a much stronger design, such as controlled interrupted-time series; however, both study designs would start off as ‘low’ certainty in GRADE.

Partly in response to these concerns, the GRADE Working Group has recently suggested a second approach, in which any body of evidence may receive a ‘high’ *initial* certainty rating provided that a rigorous tool has been used to assess risk of bias.^40^ The new risk of bias in non-randomised studies (ROBINS-I) tool for cohort-type studies is the only tool recognised by the GRADE Working Group as meeting this standard for NRSs.^40 41^ Compared with other tools, ROBINS-I offers a rigorous process for assessing risk of bias through seven distinct domains and overall 34 signalling questions, but requires both significant human resources and substantial epidemiological expertise.^41 42^ This nuanced assessment of risk of bias in ROBINS-I, specifically, regarding the domains of confounding and selection bias, allows for RCTs and NRSs to be placed on a common metric for risk of bias. While this approach enables one to initially rate evidence from NRS designs as ‘high’ certainty, it is expected that the certainty of evidence will eventually be downgraded for risk of bias, so that the final rating is the same no matter the starting point (ie, whether a body of evidence from NRSs was initially rated as ‘low’ certainty and subsequently rated up or down or whether it was initially rated as ‘high’ certainty and further downgraded). It is important to note that the current version of ROBINS-I is primarily designed for cohort studies. Although future initiatives may develop extensions to ROBINS-I for other types of NRS designs, following the original GRADE guidance for initial rating of evidence based on study design, the body of evidence comprised of NRSs other than cohort studies should be initially rated as ‘low’ certainty.^40 43^ Authors should then explain their decisions for further downgrading or upgrading of the certainty of evidence (eg, subsequent downgrading the certainty of a body of evidence from cross-sectional studies for additional concerns over the risk of bias).

### Applying GRADE domains for rating certainty

We further highlight how reviewers and guideline developers in global health may address sources of complexity when making judgements on specific domains of the GRADE approach.

#### Risk of bias

Handling performance bias in certainty ratings has proven challenging in reviews of interventions, where it is often impossible to blind participants and/or providers.^44 45^ A common source of complexity is the contingency of intervention effects on recipients’ and providers’ agency.^46^ The challenge therefore is to assess if the lack of blinding introduces a risk of bias that implies reduced confidence in the effect estimates. To do so, review authors should be careful to differentiate between ‘lack of blinding’ and the judgement for the potential of ‘performance bias’ typically associated with the lack of blinding.^47^ Lack of blinding does not always cause sufficient bias to warrant downgrading for risk of bias. Indeed, lack of blinding may be an essential aspect of the intervention of interest, particularly when knowledge of the presence of the intervention is an important aspect of its effectiveness as in a traffic safety enforcement campaign. In these circumstances, other considerations become even more important, such as blinding of outcome assessors or the nature of the comparator.^48^ For example, for the outcome ‘quality of life’, authors conducting a review on rehabilitation for chronic obstructive pulmonary disease did not downgrade for lack of blinding of provider and participants because they judged the procedures used in included studies to blind outcome assessors sufficient to address any concerns about risk of bias.^49^ Furthermore, the potential for bias due to lack of blinding will also depend on the PICO question of the review.^48^ Subjective outcomes are more prone to bias than objectively measured outcomes, such as all-cause mortality. Lack of blinding would be a more important source of bias when the comparator in the review is ‘usual care’ than an active intervention, such as when comparing two different educational interventions.

It is worth noting that assessment of performance bias has been revised in the new Cochrane tool to assess risk of bias in randomised trials (RoB V.2.0)^42^ and in ROBINS-I.^41^ In this revised version of the RoB tool, performance bias is assessed under the domain of ‘bias due to deviations from intended interventions’, which allows for assessing two different aims of the trial: either the effect of assignment to intervention or the effect of starting and adhering to intervention. When interest is in the effect of assignment to intervention (also known as ‘treatment offer’), lack of blinding of intervention recipients and providers may not warrant downgrading certainty of evidence, as the deviations from the intended intervention should not create a prognostic unbalance between the trial groups. However, in relation to starting and adhering to the intervention, deviations, such as poor adherence, poor implementation and cointerventions, may lead to risk of bias.^42^ Many interventions in global health, such as educational and behaviour change interventions, require tailoring to specific contexts. Accordingly, authors should exercise judgement on the level of differences in intervention implementation that are beyond what would otherwise be expected in a real-world context.

#### Inconsistency

Interventions examined in global health reviews often vary in how they are implemented in different contexts and in outcome measures used across settings.^3 18^ Consequently, reviews often find considerable heterogeneity in effect estimates. The proper consideration of sources of complexity when framing the review questions can facilitate assessments of whether to downgrade for inconsistency at this later stage of the review. Namely, review authors can group and synthesise the included studies according to the nature of relevant sources of complexity and, if these sources of complexity help explain heterogeneity, provide separate certainty ratings for each of these groupings.^32 50^

Judgements of inconsistency in the magnitude or direction of effects should correspond with the chosen threshold or range that the review team adopts for rating certainty of evidence. For example, if the review team chooses the null effect as a threshold for rating certainty in the estimate of effect, then judging inconsistency in the direction of effect (ie, beneficial or harmful) would be a relevant approach to follow. In this case, variation in point estimates and statistically significant heterogeneity may not warrant downgrading for inconsistency if the effects across studies are consistently in the same direction with respect to the null effect.^50 51^ However, if reviewers are rating their certainty in whether the average effect lies within an estimated range, such as within the CIs, then authors should consider multiple criteria for inconsistency (eg, overlap of CIs, degree of variation with respect to chosen thresholds and I^2^ and its p value), rather than using only a single statistical measure of heterogeneity.^50^

#### Imprecision

Judgements for imprecision are contingent on reviewers’ chosen thresholds for rating certainty. If the chosen threshold is the null effect, then imprecision will not be a concern to warrant downgrading evidence unless the confidence or prediction interval includes the null effect, in which case the evidence is either (1) imprecise (due to small number of events or participants) or (2) precise and the intervention does not have an effect relative to the comparator.^38^ For the latter, the confidence or prediction interval needs to be sufficiently narrow around the null effect to exclude a ‘meaningful’ effect established a priori.^38^ If clearly stated, authors rating certainty of effects within 95% CIs may omit a precision assessment.^38^ This approach, however, has not yet been used in any review and needs further testing on examples using complex health interventions (Montgomery *et al*, forthcoming).

#### Indirectness

Many of the reported challenges of judging indirectness can be addressed by specifying appropriate review questions. Important differences in the PICO elements beyond what is expected and specified in the review may weaken inferences regarding the directness of evidence. As outlined in the original GRADE guidance on indirectness, “it is however rare and usually unnecessary, for the intended populations and interventions to be identical to those in the studies, and evidence should be downgraded only if the differences are considered sufficient to make a difference in the outcome likely”.^52^

Another potential challenge relates to incompleteness of available evidence with regard to the review question. As questions for the global health audience might necessarily be broad (ie, often follow a ‘lumping’ approach), the available evidence might not address all elements in the PICO framework (eg, while the question may ask for evidence in both LMICs and high-income countries, the evidence may only be available for high-income countries). If authors suspect major differences in effect across the locations, rather than downgrading all evidence for indirectness, an alternative approach is to split the question to be able to provide direct evidence for a subset of conditions (eg, make separate certainty of evidence ratings for LMICs and high-income countries). In this case, authors may report a lack of evidence for the remaining subset of conditions (eg, LMICs) or extrapolate based on available data. In the latter case, however, reviewers may need to downgrade evidence for indirectness. Again, it is highly recommended that reviewers think about the factors that may modify intervention effects at the beginning of the review process, when scoping the review and formulating specific questions.

#### Publication bias

Many evaluations of global health interventions are published as reports, working papers or programme evaluations. If review authors suspect that eligible studies are likely published in this format (rather than in indexed scientific literature), a comprehensive multicomponent search that includes grey literature and contacting of experts is critical. In addition, authors should assess whether a substantial number of studies are sponsored by any vested industries (eg, intervention developers and representatives from industries benefiting from the status quo) or run by researchers with a potential ‘allegiance bias’ to warrant downgrading for publication bias.^53^

#### Upgrading

The criteria for *upgrading evidence* of complex interventions should follow the guidance of the GRADE Working Group,^54^ including guidance on upgrading when all types of study design are initially rated at ‘high’ certainty.^40^ In line with the GRADE guidance, upgrading criteria commonly apply when there are no major limitations in the body of evidence (such as risk of bias, inconsistency or imprecision).^54^ One special case for complex interventions involves intervention fidelity: authors may upgrade their certainty rating if (1) larger effects are found in studies with better implementation (criterion of dose–response effect) or (2) positive results are found among studies with low implementation fidelity (counteracting plausible residual confounding).

### Future work for rating certainty in reviews using a complexity perspective

**Figure 1:**
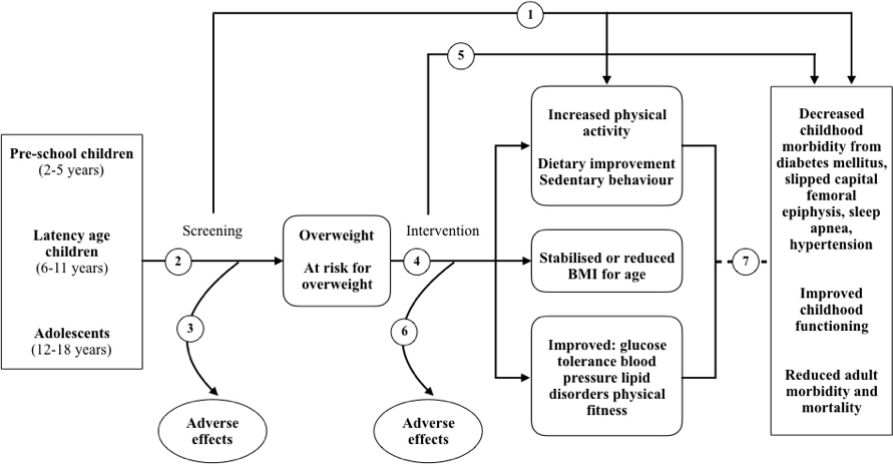
Example chain of evidence approach: screening and interventions for overweight in childhood. Arrow 1: Is there direct evidence that screening (and intervention) for overweight in childhood improves age-appropriate behavioural or physiological measures or health outcomes? Arrow 2: (1) What are appropriate standards for overweight in childhood, and what is prevalence of overweight based on these? (2) What clinical screening tests for overweight in childhood are reliable and valid in predicting obesity in childhood? (3) What clinical screening tests for overweight in childhood are reliable and valid in predicting poor health outcomes in adulthood? Arrow 3: What are the adverse effects of screening, including labelling? Is screening acceptable to patients? Arrow 4: (1) Do weight control interventions lead to improved intermediate outcomes? (2) What are common behavioural and health system elements of efficacious interventions? (3) Are there differences in efficacy between patient subgroups? Arrow 5: Do weight control interventions lead to improved health outcome and/or improved functioning? Arrow 6: What are the adverse effects of interventions? Are interventions acceptable to patients? Arrow 7: Are improvements in intermediate outcomes associated with improved health outcomes? (Only evaluated if there is no direct evidence for link 1 or link 5 and if there is sufficient evidence for link 4). BMI, body mass index. Taken from Whitlock *et al*, 2005.^57^

Based on our project findings, we suggest several areas of future work. Many interventions and complex technologies may have long and variable causal pathways. Our consultation with stakeholders suggests a strong interest in developing a robust domain for an approach to rating certainty that is based on the ‘coherence of the causal pathway’ or ‘chain of evidence’.^55 56^ A similar approach is currently used by the US Preventive Services Task Force to describe different links in the causal chain of an intervention and inform what types of evidence should be searched for and synthesised (figure 1).^57^ If review authors manage to populate different links in the causal chain of an intervention with rigorous evidence (eg, links 4 and 7 in figure 1), then this may increase their certainty in the effects of its distal outcomes (eg, link 5 in figure 1). As discussed in the previous example on deworming interventions, logic models (also known as analytical frameworks) that visually depict the links in these causal pathways can be useful in identifying the important items of evidence that should be searched for and synthesised in a systematic review.^33^ Authors could revisit their initial logic models at predefined stages of the review process, in particular, at the end of the review, using the evidence collected and synthesised for each individual link in the pathway, to assess coherence in the causal pathway originally proposed.^58^ This chain of evidence approach might be particularly informative in circumstances where direct evidence linking the intervention with the distal outcomes is unavailable. The work is ongoing both within the GRADE Working Group and beyond, for example, on how to conduct model-driven synthesis of evidence.^59^

Second, several stakeholders are interested in more systematically examining whether there are specific NRS designs that are consistently sufficiently robust to start as ‘moderate’ rather than ‘low’ certainty in the traditional GRADE approach. Such an approach would allow for a ‘quick and dirty’ sorting of study designs, where stronger NRS designs would start off as ‘moderate’ certainty and weaker designs as ‘low’ certainty. Advantages would be a quicker and more user-friendly distinction between different levels of certainty, which is likely to be applicable by many Cochrane and non-Cochrane systematic reviewers; the initial rating could then be refined by risk of bias assessments, but would be less dependent on the use of a very sophisticated risk of bias tool, such as the ROBINS-I tool. The significant challenge, however, relates to which study designs and associated features merit starting in one category versus the other.^60 61^ Based on the results of our Delphi process and the consensus meeting, this option was generally supported by those concerned with inconsistent implementation of NRS risk of bias tools leading to overestimation of certainty, especially given the novelty of the tools and varying levels of expertise in using them.^62^ However, we do not recommend this approach until future research conclusively establishes such a set of acceptable NRS designs.

## Conclusion

This primer provides a concise discussion of how to incorporate a complexity perspective when applying the GRADE approach in systematic reviews estimating the effects of interventions in global health. Key considerations include: sources of complexity when framing the review questions, such as important dimensions of context and implementation and other potential mediators and moderators of effect; a choice of a threshold or a range that matches the needs of intended users of their review, assessment of evidence from NRS designs and the criteria within each GRADE domain for rating certainty (see table 3). Suggested future work involves investigating the feasibility of (1) a domain on the coherence of evidence across the hypothesised causal pathway of an intervention, which may not need to be integrated into the GRADE ratings as it will apply at a higher, systematic review level beyond assessment of certainty of evidence in specific outcomes and (2) the identification of specific NRS designs that could start as ‘moderate’ rather than ‘low’ certainty. Researchers, including systematic reviewers, authors of HTA and guideline developers in global health should continue to report their experience using GRADE and this primer in reviews aiming to address sources of complexity. More examples of using GRADE are particularly needed for social interventions, interventions in LMIC contexts, assessments where meta-analysis may not be possible, in bodies of evidence with rigorous NRSs (as well as mixed bodies of evidence), and by researchers with varying degrees of experience in systematic reviewing and evidence assessment.

There is wide interest in finding ways to assess which interventions are effective and, equally, which ones are relevant and appropriate in diverse contexts. Using a complexity perspective can contribute to this. This discussion on considering the ‘complexity perspective’ in GRADE ratings provides a primer for systematic reviewers, authors of HTA and guideline developers to better assess evidence relating to complex interventions and systems, which could ultimately enhance the use of such evidence in global health policy and practice decisions.
